# COVID-19 vaccine hesitancy in people with migratory backgrounds: a cross-sectional study among Turkish- and German-speaking citizens in Munich

**DOI:** 10.1186/s12879-021-06940-9

**Published:** 2021-12-06

**Authors:** Zekeriya Aktürk, Klaus Linde, Alexander Hapfelmeier, Raphael Kunisch, Antonius Schneider

**Affiliations:** 1 Institute of General Practice and Health Services Research, School of Medicine, Technical University of Munich, Orleansstr. 47, 81667 Munich, Germany; 2 Institute for AI and Medical Informatics in Medicine, School of Medicine, Technical University of Munich, Munich, Germany; 3 Institute of General Practice, School of Medicine, Universitätsklinikum Erlangen, Erlangen, Germany

**Keywords:** SARS-CoV, Immigrants, Vulnerable populations, Inequalities, Vaccination refusal

## Abstract

**Background:**

This study aimed to investigate the knowledge, attitudes, behaviors, and COVID-19 vaccine hesitancy of people with migratory backgrounds among Turkish- and German-speaking patients in Munich.

**Methods:**

Primary outcomes were the intention to get vaccinated for COVID-19 and COVID-19 knowledge levels (25 true/false items). Other variables included demographics, attitudes to COVID-19 and vaccination (7 items), and behaviors regarding COVID-19 (7 items). The attitude and behavior questions had 5-point Likert scales. Of the 10 Turkish-speaking family physicians in Munich, six agreed to administer Turkish or German questionnaires to consecutive patients in February 2021. Furthermore, participants with either citizenship, country of origin, native language, or place of birth being non-German were categorized as “Having a migratory background.” Data from 420 respondents were analyzed.

**Results:**

Women constituted 41.4% (n = 174), the mean age was 42.2 ± 15.5 years, 245 (58.3%) preferred the Turkish questionnaire, 348 (82.9%) had a migratory background, and 197 (47.9%) intended to be vaccinated. The mean knowledge, attitude, and behavioral scores were 21.5 ± 3.2 (max = 25), 3.7 ± 0.8 (max = 5), and 4.0 ± 0.5 (max = 5). While 42.3% (n = 145) of the participants with a migratory background considered getting vaccinated, this proportion was 76.5% (n = 52) for non-immigrant Germans (Chi-square = 26.818, p < 0.001). Non-migratory background (odds ratio (OR): 3.082), high attitude scores (OR: 2.877), male sex (OR: 2.185), years of schooling (OR: 1.064), and age (OR: 1.022) were positively associated with vaccination intention.

**Conclusions:**

We suggest initiating or supporting projects run by persons or groups with immigrant backgrounds to attempt to elaborate and change their vaccination attitudes.

## Introduction

COVID-19 affected 69.3 million people globally by December 10, 2020 [1]. As of January 11, 2021, a total of 1,921,024 patients in Germany were recorded as COVID-19 positive [2]. Although Germany succeeded in keeping the first wave of the pandemic under control, the effects of the second wave continued, and arguably the third wave began when the study was conducted [3–5].

Europe has received many immigrants over the centuries. While factory workers constituted the majority earlier, the recent influx is mostly by asylum seekers escaping from war-torn countries such as Syria, Afghanistan, and Iraq. As of 2019, 21.8 million (4.9%) of the people living in the European Union (EU) were non-EU citizens [6].

Among the EU countries, Germany has become the number one immigrant receiver, the majority being composed of Turkish citizens. In 2018, around 3 million people with a migration background in Germany had family or religious roots in Turkey [7]. Furthermore, the number of asylum seekers from Turkey increased substantially after the coup attempt in 2016. The total number of 1767 asylum seekers from Turkey in 2015 increased to 5742 in 2016, 8483 in 2017, 10,655 in 2018, and 11,423 in 2019, making Turkey the fourth biggest defector exporter to Germany [8].

The dramatic increase of immigrants from different origins has become a significant public health concern in Europe [9]. Because migrants are positively selected in terms of their socioeconomic and health characteristics when compared to non-migrants in their country of origin, migrants have apparently good health (called the “healthy immigrant effect”). Although some studies have demonstrated a healthy immigrant effect [10, 11], others have shown poorer health status among immigrants than natives [12, 13], which suggests that the immigrants are comparatively healthier initially on entrance but become disadvantaged over time. Among immigrants’ health concerns is vaccine hesitancy, which is defined as a delay in acceptance or refusal of vaccines despite the availability of vaccination services [14]. It includes factors such as complacency, convenience, and confidence.

Despite the half-century history of work‐related migration, migrant populations still face particular social, economic, and health problems. This also applies to the former Turkish “guest workers” and their families in Germany. Studies are reporting that Turkish migrants in Germany require special public health attention [15]. Health service satisfaction [16] and disparities [17] are two of the reported concerns. At our Munich location, there are around 40 thousand people of Turkish origin [18], but there is no available data concerning the status of this subgroup of persons with regard to COVID-19 and vaccine hesitancy.

## Objectives

This study aimed to investigate the knowledge, attitudes, behaviors, and vaccine hesitancy regarding the COVID-19 pandemic of people with migratory backgrounds among Turkish- and German-speaking patients of Turkish-speaking family doctors in Munich.

## Methods

### Study design

An anonymous survey was conducted in a cross-sectional design. Study reporting was done per the Strengthening the Reporting of Observational Studies in Epidemiology (STROBE) guideline [19]. The study was approved by the ethics committee for the medical faculty of the Technical University of Munich (January 20, 2021, Number: 37/21 S-EB). An information letter explaining the study details was handed over to all participants. As approved by the ethics committee, no written consent was deemed necessary; filling in the questionnaires was interpreted as an agreement to participate. All methods were implemented in accordance with the ethical guidelines and regulations of the host institution.

### Setting

The study was conducted within February 2021 in Munich. Participants were German- and Turkish-speaking patients of Turkish-speaking family physicians. Munich is a city with 1.472 million citizens [20], of which around 40,000 are of Turkish origin [18].

### Participants

**Fig. 1:**
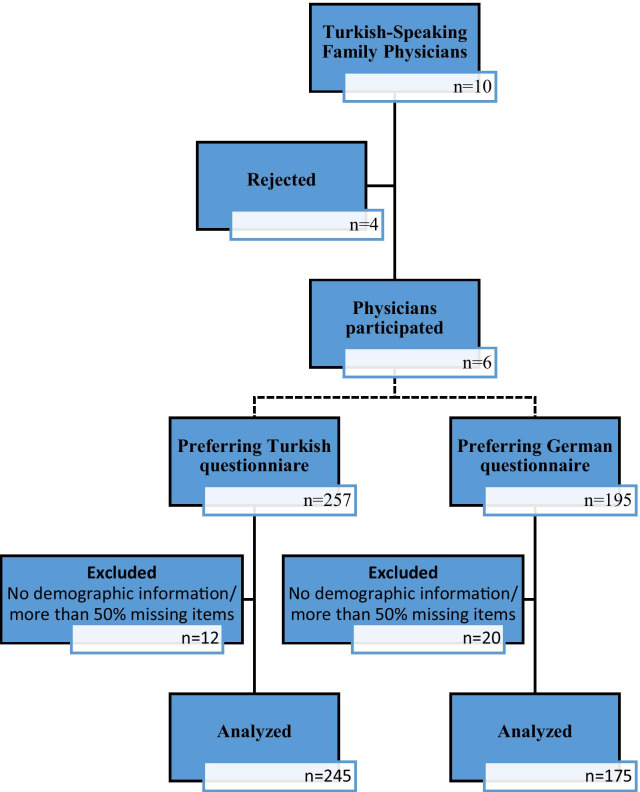
Study flow diagram

A list of the 612 family physicians working in Munich was obtained from the Bavarian Association of Statutory Health Insurance Physicians “Kassenärztliche Vereinigung Bayerns (KVB),” which was reviewed for Turkish names. Additionally, an internet search and snowball inquiry was made by contacting the Turkish-speaking family physicians, which returned a list of 10 such physicians. The principal investigator visited all targeted family doctors in their offices and inquired about their willingness to support the study. As a result, six family physicians agreed to distribute the study questionnaires to their patients. Inclusion criteria were being the age of 18 and above, sufficient knowledge of the German or Turkish language, and willingness to volunteer to participate. Participants who did not disclose their demographic information or had more than 50% missing items in the questionnaire were excluded from the analysis. A flow diagram of physician and patient participation is given in Fig. 1.

### Variables

The primary outcome measures of the study were COVID-19 knowledge scores and intention to get vaccinated for COVID-19. Other study variables were related to attitudes and behaviors regarding COVID-19 and demographic information (age, sex, citizenship, native language, place of birth, and total years of schooling).

Citizenship/nationality, native language, and place of birth were considered as additional variables for subgroups to take the complex construct of a “migration background” into account. Thus, participants with either citizenship/nationality, country of origin, native language, or place of birth being non-German were categorized as “Having a migratory background.”

### Construction of the questionnaire

The researchers developed the study questionnaire after reviewing the literature for common knowledge and guideline recommendations related to COVID-19. Two team meetings were conducted to refine the questionnaire items. In addition, a pilot test on five Turkish and five German-speaking participants was conducted to further modify the questionnaire. The final questionnaire consisted of a demographic information section (six questions) and three subdomains, including knowledge, attitudes, and behaviors concerning COVID-19.

The knowledge domain contained 25 items, which were scored as true or false. Hence, the COVID-19 knowledge scores were calculated by summing up the correct answers, providing a minimum of 0 and a maximum of 25.

The attitude and behavior domains included both seven items arranged in a five-point Likert scale (1 = disagree/never, thru 5 = agree/very frequent). Item numbers 2, 3, 5, and 6 in the attitude domain and items 4, 5, 6, and 7 in the behavior domain were reversely coded. Attitude and behavior scores were calculated by adding the scores of each item and dividing by 7 (the total number of items), which revealed the minimum and maximum possible scores of 1 and 5. Higher scores indicated more sensitive attitudes and behaviors.

The two-page paper questionnaire was made available in German and Turkish.

### Data sources/measurements

The practice personnel was asked to consecutively approach patients speaking German or Turkish and invite them to participate in the questionnaire survey for recruitment.

All interested individuals were given the patient information letter and the study questionnaire. If the individual either spoke Turkish or German (or one language much better than the other), they received the material in the appropriate language. If the individual spoke both languages, they were asked for their preference. Questionnaires were self-administered in a silent place and collected after completion by the practice personnel or the primary author.

### Data quality and bias

Each participant was asked to fill in the questionnaire alone without interference from others. After digitalizing the data, error-checking and debugging were done to eliminate questionnaires with missing data or conflicting information. In addition, an effort was given to minimize selection bias by recruiting concomitant patients. However, although the principal investigator spent time in the practices reassuring data collection according to the protocol, this was not always possible due to the local conditions and the rules and regulations discouraging prolonged stay of patients in practice and accommodating multiple patients at a time.

### Sample size

A sample size of n = 400 in the cross-sectional study was determined to be sufficient for consistent estimation of the coefficients of a multiple logistic regression model, including the factor variable ‘group’ and the demographic variables for adjustment. Further, the sample size is efficient for subgroup analyses (e.g., vaccination hesitancy, migratory background, etc.) [21].

### Statistical methods

The data were entered into the IBM SPSS Statistics spreadsheets (IBM Corp, Armonk, NY). The data distribution was described within and across the study groups by frequencies, percentages, means, and standard deviations (SD), as appropriate. Cronbach’s alpha was computed to assess the reliability of the items belonging to the knowledge, attitude, and behavior domains. Corresponding hypothesis-testing of univariable group differences was performed by Chi-squared tests, Fisher’s exact tests (or Fisher–Freeman–Halton test), and independent samples t-tests or Mann–Whitney-U tests. Multiple binary logistic regression models were fit to the data to adjust the effect estimation of group differences by potential predefined confounders, such as demographics. Significant variables affecting vaccination intention in the univariate analyses were included in the model. Hypothesis testing was performed at exploratory two-sided 5% significance levels.

## Results

### Participants and migratory backgrounds

Of the invited patients, 452 agreed to participate. Questionnaires with missing demographic data (n = 2) were excluded. Also excluded were a total of 27 participants with more than 50% missing items in the knowledge (n = 9), attitude (n = 23), or behavior (n = 20) domains, and 3 participants with missing migratory background information. Data of 420 participants were analyzed (women: 41.4% (n = 174), mean age: 42.2 ± 15.5 years) (Fig. 1).

Of the participants, 245 (58.3%) preferred the Turkish questionnaire. In addition to these 245 people, 103 (58.8%) of the 175 participants who preferred the German questionnaire had a migratory background, making 348 (82.8%). There were 72 (17.1%) German participants without a migratory background. Of the 348 patients with a migratory history, 90 (25.8%) were German nationals. Of the 71 participants with a migratory background who were born in Germany, only 42 (59.1%) had German nationality. In the case of those with migratory backgrounds, there were significant differences in terms of age, sex, and preferred language. Among the participants with migratory backgrounds, prominent countries of origin were Turkey, Bulgaria, and Iraq, while Turkish, Bulgarian, Kurdish, and Arabic were the most commonly spoken native languages (Table 1).

**Table 1:** Sociodemographic characteristics

	Migratory background				Test	p
	Yes		No			
	n/Mean	%/SD	n/Mean	%/SD		
Your sex						
Female	130	37.4	44	61.1	13.542^#^	**0.001**
Male	216	62.1	28	38.9		
Other	2	0.6	0	0		
Your age (years)	39.3	12.6	56	20.2	6.476^$^	**< 0.001**
Total years of schooling	13.1	4.5	12.6	3.4	1.411^$^	0.158
Did you have the COVID-19 infection?						
Yes	59	18.7	8	12.5	1.395*	0.237
No	257	81.3	56	87.5		
Which language do you prefer?						
Turkish	245	70.4	0	0	121.655*	** < 0.001**
German	103	29.6	72	100		
Which nationality do you have?						
Turkish	197	57.9				
German	90	26.5	72	100		
Bulgarian	26	7.6				
Other	27	7.9				
What is your country of origin						
Turkey	263	76.5				
Germany	12	3.5	72	100		
Bulgaria	28	8.1				
Other	41	11.9				
What is your native language						
Turkish	263	77.8				
German	19	5.6	72	100		
Bulgarian	12	3.6				
Kurdish	13	3.8				
Arabic	8	2.4				
Other	23	6.8				
Where have you been born?						
Turkey	207	60.7				
Germany	71	20.8	72	100		
Bulgaria	25	7.3				
Iraq	9	2.6				
Other	29	8.5				

Significant p-values are highlighted in bold

*SD* standard deviation

^#^Fisher’s exact test, *Chi-square, ^$^Mann–Whitney U test

### Descriptive findings and outcomes

Responses to the scale items are summarized in Table 2. On average, the knowledge level regarding COVID-19 was high, but looking at attitudes revealed that there was a relatively low fear of death due to the disease. Also, very few participants thought that faith would play a role in protection from the disease. Behavior scores, on the other hand, were comparatively higher (Table 2). Cronbach’s alpha reliability coefficients for the knowledge, attitude, and behavior domains were 0.732, 0.695, and 0.716, respectively.

**Table 2:** Domains and descriptive statistics of the survey items

Subscale 1: COVID-19 knowledge	Correct n (%)	False n (%)
1. The cause of the Corona-infection a virus	353 (87.4)	51 (12.6)
2. How COVID-19 spreads is not known	287 (69.5)	126 (30.5)
3. COVID-19 can spread through the air in enclosed spaces	388 (93.3)	28 (6.7)
4. COVID-19 can spread through close contact (e.g. hugging)	394 (93.8)	26 (6.2)
5. COVID-19 can spread through sexual contact	242 (59.9)	162 (40.1)
6. COVID-19 is often transmitted through food	333 (80.2)	82 (19.8)
*Which of the following measures can reduce the risk of transmitting COVID-19?*	408 (96.5)	15 (3.5)
7. Washing hands after touching potentially infected surfaces	412 (97.4)	11 (2.6)
8. Wearing a face mask when entering crowds	368 (90.2)	40 (9.8)
9. Taking antibiotics	335 (80.5)	81 (19.5)
10. Drinking vinegar	363 (87.5)	52 (12.5)
11. Drinking carrot juice	405 (96)	17 (4)
12. Keeping a distance of 1.5 m from people	400 (95.7)	18 (4.3)
13. Lubricate butter in the nostrils	311 (75.1)	103 (24.9)
14. Eating garlic	222 (54.8)	183 (45.2)
15. The use of the Corona app	401 (94.8)	22 (5.2)
16. Frequent ventilation when in the same room with others	374 (88.6)	48 (11.4)
17. Avoiding closed rooms with strangers	397 (94.3)	24 (5.7)
18. Avoiding crowds	382 (91)	38 (9)
19. Drinking holy water	394 (94.3)	24 (5.7)
*Which of the following symptoms are common in COVID-19?*	407 (96.9)	13 (3.1)
20. Cough	363 (89.4)	43 (10.6)
21. Fever	395 (96.8)	13 (3.2)
22. Dysuria	387 (95.3)	19 (4.7)
23. Increased appetite	391 (93.8)	26 (6.2)
24. Weight gain	287 (69.5)	126 (30.5)
25. Loss of taste and smell	388 (93.3)	28 (6.7)

Subscale 2: COVID-19 attitude 5-Agree, 4-Partially agree, 3-Not sure, 2-Partially disagree, 1-Disagree	Mean	SD
1. COVID-19 is dangerous	4.47	1.04
2. In reality, COVID-19 does not exist	1.63	1.24
3. The danger of COVID-19 is exaggerated	2.42	1.52
4. I am afraid of dying if I should get COVID-19	2.50	1.45
5. Believers are protected from COVID-19	1.47	1.12
6. COVID-19 was created purposely to control the world	2.47	1.50
7. Vaccination against COVID-19 is safe	3.19	1.29

Subscale 3: COVID-19 behavior 1-Never, 2-Very rare, 3-Off and on, 4-Frequent, 5-Very frequent	Mean	SD
1. How often do you wash your hands?	4.38	0.64
2. How often do you wear a mask when you are outside?	4.19	0.90
3. How much attention do you pay to keeping distance?	4.16	0.88
4. How often do you accept guests?	2.13	0.88
5. How often do you go visiting others?	1.82	0.91
6. How often do you enter crowded places?	2.05	0.91
7. How often do you use public transport?	2.27	1.23

	Yes n (%)	No n (%)	Not sure n (%)
Have you already been vaccinated against COVID-19?	23 (5.5)	397 (94.5)	
Will you get vaccinated against COVID-19?	198 (47.8)	77 (18.6)	139 (33.6)
Why? Can you please elaborate?			

A total of 197 participants (47.9%) intended to get vaccinated. However, there were significant differences in the mean knowledge, attitude, and behavior scores associated with a migratory background. Most participants with a migratory background were not planning to be vaccinated and had lower scores in all three subscales. However, COVID-19 knowledge scores were relatively high for both groups (Table 3).

**Table 3:** Descriptive statistics and comparisons between the migratory backgrounds

	Migratory background						Test	p
	Yes (n = 348)		No (n = 72)		Total (n = 420)			
	n	%	n	%	n	%		
Have you already been vaccinated against COVID-19?								
Yes	16	4.6	7	9.7	23	5.5	3.027^#^	0.091
No	332	95.4	65	90.3	397	94.5		
Will you get vaccinated against COVID-19?								
Yes	145	42.3	52	76.5	197	47.9	26.818^*^	**< 0.001**
No	70	20.4	7	10.3	77	18.7		
Not sure	128	37.3	9	13.2	137	33.3		

	Mean	SD	Mean	SD	Mean	SD		
Knowledge score	21.2	3.4	23.3	1.7	21.6	3.2	5.660^$^	**< 0.001**
Attitude score	3.6	0.8	4.2	0.7	3.7	0.8	5.317^$^	**< 0.001**
Behavior score	4.0	0.6	4.3	0.5	4.1	0.6	3.715^&^	**< 0.001**

Significant p-values are highlighted in bold

*SD* standard deviation

^*^Chi-square, ^#^Fisher’s exact test, ^&^Independent samples t-test, ^$^Mann–Whitney U test

**Fig. 2:**
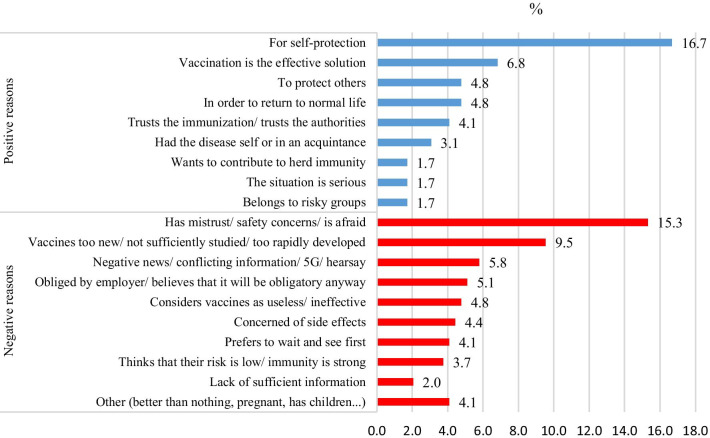
Distribution of the reasons for accepting or rejecting vaccination

Of the 411 patients who indicated their intentions to either accept or refuse vaccination, 253 (61.5%) expressed 1–3 reasons for their thoughts (a total of 294). After categorization of the free texts, the three most common reasons were self-protection (n = 49), concerns about safety or mistrust in vaccines (n = 45), and the perception that vaccines were not sufficiently studied (n = 25) (Fig. 2).

Of the people with a migratory background, 12.6% (n = 44) agreed or somewhat agreed with the statement that COVID-19 actually does not exist. This belief was held by only 5.6% (n = 4) among the non-immigrant participants (Chi-square = 11.623, p = 0.020). Also, the number of participants believing that the COVID-19 was purposely created to control the world was higher among respondents with a migratory background (30.6% of migrant applicants (n = 106) agreed or somewhat agreed vs. 5.6% (n = 4) of non-migrants) (Chi-square = 33.020, p < 0.001).

### Multivariable analyses

Responses to the question “Will you get vaccinated against COVID-19?” were organized into two categories namely “No + Not sure” and “Yes.” Positive vaccination intentions ranged from 26.9 to 68.5% between different practices.

All investigated variables were related to the vaccination intention. It was also noted that more participants with a preference for a German questionnaire had vaccination intentions than those preferring a Turkish questionnaire (n = 99/57.9% vs. n = 99/40.6%, Chi-square = 12.091, p = 0.001), and men had higher intentions to get vaccinated compared to women (Table 4).

**Table 4:** Univariate analyses and multiple logistic regression model concerning the agreement for COVID-19 vaccination

	Univariate comparisons									Multiple logistic regression model					
	n/Mean	%/SD	n/Mean	%/SD	Wald	p	OR	95% CI		B	Wald	p	OR	95% CI	
								Lower	Upper					Lower	Upper
Age (years)	37.7	13.5	46.5	15.8	30.745	**< 0.001**	1.042	1.027	1.057	0.022	4.459	**0.035**	1.022	1.002	1.042
Sex (male)	112	46.3	130	53.7	8.608	**0.003**	1.817	1.219	2.707	0.782	8.256	**0.004**	2.185	1.282	3.725
Total years of schooling	12.3	4.1	13.7	4.5	10.9	**0.001**	1.086	1.034	1.14	0.062	3.582	0.058	1.064	0.998	1.134
Migratory background (no)	16	23.5	52	76.5	23.705	**< 0.001**	4.438	2.436	8.085	1.125	6.767	**0.009**	3.082	1.32	1.195
Was infected with COVID-19 (no)	150	48.2	161	51.8	4.446	**0.035**	1.813	1.043	3.152	0.472	2.003	0.157	1.603	0.834	
Knowledge score	20.9	3.5	22.1	2.8	11.525	**0.001**	1.121	1.049	1.192	-0.029	0.364	0.546	0.971	0.883	1.068
Attitude score	3.4	0.8	4.1	0.6	62.542	**< 0.001**	3.762	2.709	5.224	1.057	23.393	**< 0.001**	2.877	1.875	4.414
Behavior score	3.9	0.6	4.2	0.4	26.018	**< 0.001**	2.841	1.902	4.243	0.296	1.111	0.292	1.344	0.776	2.33
Constant										-6.117	19.508	**< 0.001**			

Significant p-values are highlighted in bold

*SD* standard deviation, *CI* confidence interval, *OR* odds ratio

A multiple logistic regression model was fit to the data to estimate and test the relation of predictors of vaccination intention. Independent predictors of the model included sex (male/female), migratory background (yes/no), age (years), duration of schooling (years), a previous infection (yes/no), knowledge score, attitude score, and behavioral score.

The multivariable analysis changed the significance levels. Age, male sex, years of schooling (borderline significant), migratory background, and high attitude scores positively affected the vaccination intention (Table 3). The most critical variable was the migratory background with an odds ratio (OR) of 3.1, followed by attitude scores (OR 2.9) and sex (OR = 2.2).

## Discussion

### Key results

This study found significant differences among patients with and without migratory backgrounds in the studied sample concerning COVID-19. While 82.8% had a migratory background, 42.1% preferred the German questionnaire. Furthermore, while 42.3% of the participants with a migratory background were considering becoming vaccinated, this proportion was 76.5% for non-immigrant Germans. On the other hand, this group’s mean knowledge, attitude, and behavioral scores were relatively high. After correcting for potential confounders, migratory background, increased attitude scores, male sex, longer schooling, and higher age positively affected vaccination intention.

### Limitations

This study approached patients of Turkish-speaking general practitioners (GPs) in Munich. Hence, its interpretation should consider this context. Slight differences could be expected if the sample had been selected from all GPs, including native German physicians, which might limit the generalizability of the results. As another potential limitation, we may mention that the survey questionnaire was not validated before. However, as described in “Methods” section, an effort was given to standardizing the items. Furthermore, all three subscales revealed reasonable internal consistency findings.

### Interpretation

People originating from Turkey constitute the highest proportion of citizens with foreign origin in Germany, followed by Poland and Syria [22]. However, they often find themselves not so highly appreciated and accepted [23, 24]. Considering the participants’ mean age of 42.2 years, attention is needed for those with immigrant backgrounds and who were born in Germany but still did not obtain German citizenship. The relatively high proportion of this group (40.9%) confirms a lack of successful integration even in the third generation.

Although there was a recent influx of immigrants from Turkey (around 40,000 within 5 years) [8], they constitute a minor proportion; the majority are the offspring of the first immigrants after 1961 [7]. Hence, the healthy immigrant effect [10, 11] seems to be negligible here. Our study demonstrated significant shortcomings among people with migratory backgrounds concerning the intention to be vaccinated as well as knowledge, attitudes, and behaviors concerning COVID-19. Thus, interventions are highly required to close the health-related gap in this group of citizens. Besides the efforts of the German state, projects initiated by the Turkish-speaking community may accelerate the integration process [25].

People of Turkish origin make up the largest migration group in Germany, so their different social, political, and health aspects have been widely studied. Therefore, we would like to present some more information on the situation of Turkish migrants and their descendants in Germany to understand the broader social dynamics that could explain the distrust of vaccines. This segment of the German population experiences particular social disadvantages and displays a decline in some aspects of health across generations [26]. Examples of health-related disadvantages include the lack of infrastructure for religious practices, gender-specific treatment options, and nutritional preferences in hospitals [27]. In addition, policies such as the test for Muslim citizenship applicants [28] may have contributed to the introversion of Turkish immigrants and their rejection of Western values.

People’s acceptance of immunization against COVID-19 was a concern even before specific vaccines became available. In particular, nursing staff in Germany have been reported to be hesitant to get vaccinated. Although they are in the highest priority group, only 46.6% had been vaccinated as of April 2021 [29]. It is worth mentioning that 23.2% of these healthcare workers have a migratory background [30].

Presently 70% of the German population is expected to accept the COVID-19 vaccination [31]. Disagreements among health professionals and politicians [32] as well as fake news [33] account for a substantial part of the confusion in public. However, research shows a high degree of hesitation rather than direct opposition to COVID-19 vaccination [33], which is similar to the results from our case: Of the 52% who intended not to be vaccinated or were not sure, 33.3% were hesitant to make a decision, while 18.7% were refusers. At this point, the reasons for vaccine refusal should be noted.

Access to healthcare was proposed as another barrier to vaccination, especially for unregistered immigrants [34]. Lack of access combined with a lack of information about the contents and side effects of the vaccine could lead to a low perceived need, causing vaccine hesitancy. Therefore, hesitation should be addressed with clear, accessible, and individualized information campaigns. Furthermore, ethnic minorities and insecure migrant populations have low intent and uptake of vaccine services [35]. The mistrust might have its origins in the sporadic racist attacks on immigrants, particularly those with Turkish roots. From this perspective, involving trainers from the same community could increase the possibility of acceptance and help people embrace the information that is transferred.

The proportion of participants with an immigrant background who refused vaccination in our study was similar to figures reported from Turkey. In a study conducted on 272 women and 156 men, 66.1% considered not getting vaccinated against COVID-19, and women were less likely to be willing to get a vaccine than men [36]. Lower vaccination rates among women compared to men have been reported by other researchers [37].

As to our findings regarding the motives for accepting or rejecting vaccination, it can be speculated that the rapid developmental process of the COVID-19 vaccines and the global immunization campaigns without satisfying information transfer to the public has contributed to the concerns about the safety of vaccines or raised mistrust in vaccines and the perception that vaccines have not been sufficiently studied. Looking at the distributions of the reasons for accepting or rejecting vaccination in our study, negative news, conflicting information, and conspiracy theories rank in third place. Of the people with a migratory background, 12.6% believed that COVID-19 does not exist, compared to 5.6% among the non-immigrants. An even higher number of immigrants (30.6%) assumed that COVID-19 was purposely created to control the world.

Combining the above information with integration tardiness, we may deduct that Turkish immigrants in Germany are substantially affected by external negative factors. It was reported that 6.17% of adults in Western Turkey received no lifetime vaccination at all and anticipated a further decline in vaccination rates [38]. In fact, vaccine hesitancy proportions in Turkey are growing dramatically. The number of families who signed a vaccine rejection form increased from 183 in 2011 to 12,000 in 2016 and 23,650 in 2017 [39]. As of the 12th of September 2021, the complete (two doses) vaccination rates of Germany and Turkey were 62.3% and 49.6%, respectively [40]. The comparatively lower vaccination readiness of the Turkish immigrants in Germany might be attributed to experiences of discrimination and racism as well as a mistrust in public authorities. Although it is possible that Turkish migrants are exposed to information sources from Turkey rather than Germany, it could also be that they are more exposed to the type of German information sources that spread misinformation on vaccines because they rely more on social media and have less trust in the government.

Bearing in mind that a significant number of participants were critical or suspicious against vaccination, the relatively high knowledge scores suggest an informed decision. Participants scored 21.5 points out of a maximum of 25. In other words, those who are against COVID-19 vaccination or have reservations devised their opinions possibly not because of a lack of knowledge but rather due to their attitudes shaped by factors not addressed in our study. A multivariate analysis endorses this conclusion. After correcting for potential confounders, the most significant two variables affecting the decision to be vaccinated were the migratory background (OR 3.1) and attitude scores (OR 2.9). Qualitative studies could ascertain the reasons behind the negative attitudes concerning the COVID-19 vaccination.

## Conclusion

Significant differences exist between patients with and without migratory backgrounds of Turkish-speaking family physicians in Munich. We conclude that the negative attitudes of people with a migratory background toward COVID-19 contribute to their decisions not to be vaccinated. Additionally, vaccination refusal in the studied population depends on intertwined factors, including migratory background, age, sex, educational status, and attitudes toward COVID-19. More effort is needed to integrate and equalize the immigrants in Germany with the native community. Only then can this disadvantaged population group be protected from unnecessary health risks, which means protecting the whole population via achieving herd immunity by vaccination. Specifically, we suggest initiating or supporting projects run by persons or groups from among the immigrants. This will hopefully contribute to the ownership and success of the interventions. Furthermore, qualitative and mixed-methods studies could shed more light on the issue by conducting an in-depth investigation of the relevant factors affecting the negative attitudes.

## Data Availability

The datasets used and/or analyzed during the current study are available from the corresponding author on reasonable request.
